# Bile Acids and the Microbiome: Making Sense of This Dynamic Relationship in Their Role and Management in Crohn's Disease

**DOI:** 10.1155/2022/8416578

**Published:** 2022-03-22

**Authors:** Aditi Kumar, Hafid O. Al-Hassi, Helen Steed, Oliver Phipps, Matthew J. Brookes

**Affiliations:** ^1^Department of Gastroenterology, The Royal Wolverhampton NHS Trust, New Cross Hospital, Wolverhampton, UK; ^2^School of Medicine and Clinical Practice, Faculty of Sciences and Engineering, University of Wolverhampton, Wolverhampton, UK

## Abstract

**Background:**

Bile acids help maintain the physiological balance of the gut microbiome and the integrity of the intestinal epithelial barrier. Similarly, intestinal bacteria play a major role in bile acid metabolism as they are involved in crucial biotransformation steps in the enterohepatic circulation pathway. Understanding the relationship between bile acid signalling and the gut microbiome in Crohn's disease can help target new and innovative treatment strategies.

**Aims:**

This review summarises the relationship between bile acids and the microbiome in Crohn's disease and discusses potential novel therapeutic options.

**Methods:**

We performed a literature review on bile acid signalling, its effect on the gut microbiome, and therapeutic applications in Crohn's disease.

**Results:**

Current research suggests that there is a strong interplay between the dysregulated microbiota, bile acid metabolism, and the mucosal immune system that can result in a changed immunological function, triggering the inflammatory response in Crohn's disease. Recent studies have demonstrated an association with altering the enterohepatic circulation and activating the farnesoid X receptor signalling pathway with the use of probiotics and faecal microbial transplantation, respectively. Bile acid sequestrants have been shown to have anti-inflammatory, cytoprotective, and anti-apoptotic properties with the potential to alter the intestinal microbial composition, suggesting a possible role in inducing and maintaining Crohn's disease.

**Conclusions:**

Active Crohn's disease has been correlated with changes in bacterial concentrations, which may be associated with changes in bile acid modification. Further research should focus on targeting these areas for future therapeutic options.

## 1. Introduction

**Figure 1:**
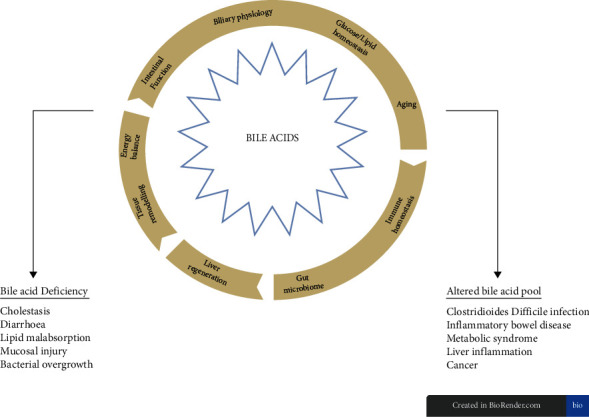
The multifunctional roles of bile acids and the symptoms and diseases they can contribute to when the BA pool is deficient or altered. Image modified from Perino et al. [11].

Bile acids (BA) are the main components of human bile and have an integral role in maintaining health, specifically through the absorption of nutrients and vitamins [1]. BA also have immunomodulatory actions and appear to be a major regulator of the gut microbiota [2]. They also have extensive roles in glucose homeostasis, lipid and lipoprotein metabolism, energy expenditure, intestinal motility and bacterial growth, inflammation, liver regeneration, and hepatocarcinogenesis (Figure 1) [3]. BA deficiency can cause cholestasis, diarrhoea, lipid malabsorption [4] and, in the intestine, is associated with mucosal injury and bacterial overgrowth [1]. An altered BA pool is associated with several disease states, including but not limiting to recurrent *Clostridioides difficile* infections (rCDI) [5], inflammatory bowel disease (IBD) [6], metabolic syndromes (such as diabetes) [7], liver inflammation (such as nonalcoholic fatty liver disease and primary sclerosing cholangitis) [8, 9], and cancers (such as colorectal cancer) (Figure 1) [10]. This review paper will focus on the impact of BA and the microbiome, and explore potential novel targets for the treatment of Crohn's disease (CD), a subcomponent of IBD.

## 2. Review Criteria

A literature search was conducted on PubMed with relevant papers on BA signalling used without publication date restrictions. For general knowledge and well-established literature on BA physiology, review articles from high-impact journals were cited. Regarding experimental and clinical studies, both animal and human trials were included and stated accordingly in the text. Peer-reviewed publications in English only were taken into consideration. This review is not designed as a systematic review.

## 3. Bile Acids

BA are derived from cholesterol to form primary BA, namely, cholic acid (CA) and chenodeoxycholic acid (CDCA) [12]. Primary BA are then conjugated with glycine or taurine amino acids, which increase their solubility, and stored in the gallbladder prior to excretion from the biliary ducts [6, 13]. Conjugation has several benefits, including minimising passive absorption [14], modulating host inflammatory responses, and regulating the gut microbiota [12]. The relatively high concentration of conjugated BA in the small intestinal lumen may play an important factor in the paucity of microbes in this area as they can inhibit the growth of bacteria in the small intestine mediating an antimicrobial effect [15].

BAs are released from the gallbladder into the small intestine postprandially in response to the hormone, cholecystokinin (CCK) [16]. Once in the small intestine, BAs emulsify dietary fat and enhance lipid, sterol, and vitamin absorption [16]. Most of the BA remain in the gut lumen until they reach the TI. Approximately 95% of BA will be actively reabsorbed in the ileum via the apical sodium-dependent bile acid transporter (ASBT). In the cytoplasm of the enterocyte, BA will bind to the ileal bile acid binding protein (IBABP), which will allow them to be excreted into the portal circulation by the organic anion transporter polypeptide (OATPA/B). This receptor is found on the basolateral membrane of enterocytes. The BA will travel back into the liver through the sodium taurocholate cotransporting polypeptide (NTCP) transporter [16, 17]. Once in the liver, free BA is reconjugated with taurine or glycine before secretion into the biliary tract and intestinal lumen [12]. This metabolic loop constitutes the enterohepatic cycle of BA [6] and occurs 8–10 times per day [16]. This recycling is necessary as hepatocytes have limited capability to produce BAs [18].

**Figure 2:**
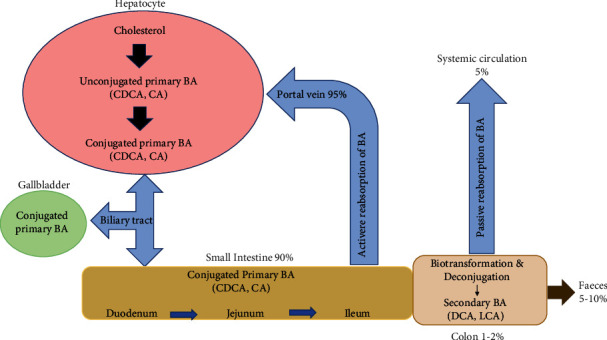
The enterohepatic circulation of bile acids (BA). Primary BA are synthesised from cholesterol in the liver and subsequently conjugated to taurine or glycine, which increases their solubility. The conjugated primary BA are then stored in the gallbladder until they are released into the small intestine by the hormone cholecystokinin (CCK) after a meal. BAs will travel through the small intestine aiding in the digestion and absorption of lipids and fat-soluble vitamins. They will be actively reabsorbed in the ileum where they will return to the liver via the portal vein. A small proportion of BAs will escape intestinal reabsorption and enter the colon. In the colon, the resident gut microbiota will promote the deconjugation and biotransformation of the primary BAs into the secondary and more hydrophilic BAs, deoxycholic acid (DCA) and lithocholic acid (LCA). The majority of secondary BA will re-enter the systemic circulation via passive reabsorption, and a small amount (5%) will be excreted in the faeces.

**Figure 3:**
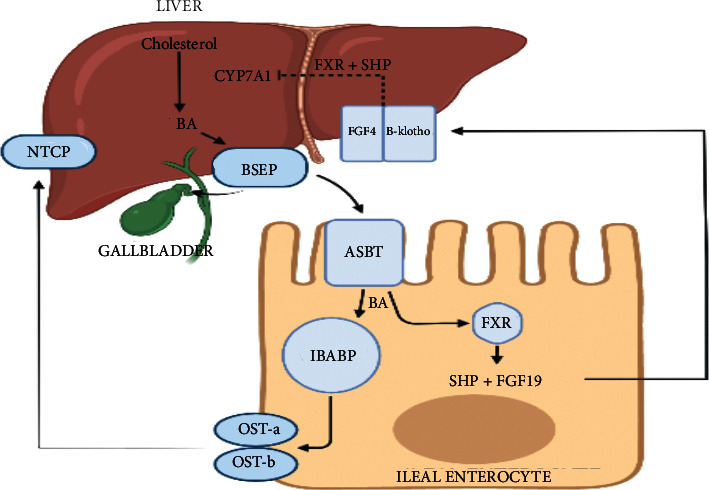
Bile acid signalling within the enterocyte. Bile acids are formed from the breakdown of cholesterol via the CYP7A1 enzyme. Bile acids are then transported from the liver through the bile salt export pump (BSEP). They will then travel via the biliary tree to the gallbladder for storage. After a meal, bile acids are then ejected into the small intestine where they are actively absorbed into the brush border of the terminal ileal epithelial cell through the apical bile acid transporter (ASBT). In the cytoplasm of the enterocyte, bile acids bind to the ileal bile acid binding protein (IBABP) and then are excreted via the basolateral heterodimeric protein OST alpha and beta. Bile acids enter the portal venous circulation and return to the liver via the Na+-taurocholate polypeptide (NTCP). In the enterocyte and hepatocyte, bile acids will bind to FXR-activating FGF19 and SHP. These proteins will then travel to the liver to create a negative feedback pathway and inhibit further bile acid synthesis (image created by https://www.biorender.com).

Ileal BA transport is highly efficient, but a small proportion (1-2%) of BA will escape the enterohepatic circulation and enter the large intestine [19]. As they transit through the colon, the microbiota will perform several enzymatic reactions, namely, deconjugation, dihydroxylation, and epimerisation, to form secondary BA: deoxycholic acid (DCA) from CA and ursodeoxycholic acid (UDCA) and lithocholic acid (LCA) from CDCA [6, 20]. Deconjugation allows BA to undergo further modifications by the intestinal microbiota and is thus crucial in bile biotransformation [21]. This metabolism of BA by the microbiota makes them more lipophilic, enabling the secondary BA to be reabsorbed passively in the large intestine and transported back to the liver via the systemic circulation [6]. See Figures 2 and 3 for a breakdown of the enterohepatic circulation and BA signalling pathway.

### 3.1. Farnesoid X Receptor (FXR)

The farnesoid X receptor (FXR) is a nuclear hormone receptor found in enterocytes that binds to BA and is a key regulator in BA metabolism [12, 20]. FXR is known to be part of a superfamily of nuclear receptors [22]. Nuclear receptors are ligand-activated transcription factors that regulate development, reproduction, and metabolism through the response to lipophilic ligands such as hormones, vitamins, and dietary lipids [22]. FXR is mainly expressed in the ileum and liver, but can also be found in the kidneys and adrenal glands [23]. FXR can be activated by either free or conjugated BAs but has a stronger binding affinity towards CDCA and less so with LCA, DCA, and CA, whilst UDCA and hydrophilic BAs are unable to activate FXR [18]. After binding with BAs, FXR attaches to the retinoid X receptor (RXR) to form a heterodimer, which can then regulate gene transcription involved in BA synthesis, transport, and metabolism in the liver and intestine [22, 24]. FXR can also activate the angiogenin (Ang1) gene and the nitric oxide synthase (iNos) genes that are involved in enteric protection and inhibition of bacterial overgrowth [21]. FXR activity alleviates inflammation and preserves the intestinal epithelial barrier by regulating the extent of the inflammatory response, maintaining the integrity and function of the intestinal barrier, preventing bacterial translocation into the intestinal tract, and regulating the growth of the microbiota [18].

### 3.2. Fibroblast Growth Factor 19 (FGF19)

The most FXR-responsive protein in the human ileum is the fibroblast growth factor 19 (FGF19) and, in rodents, the fibroblast growth factor 15 (FGF15) [20]. FXR stimulates the production and secretion of FGF19, which then binds to the surface FGF receptor 4 associated with the b-klotho (KLB) protein [25]. This ligand-receptor complex activates a mitogen-activated protein cascade that then inhibits the activity of CYP7A1, the enzyme that initiates BA synthesis from cholesterol [26]. Thus, the activation of FGF19 results in reduced new BA synthesis [27]. This feedback mechanism ensures the regulation of BA synthesis such that if sufficient amounts of BA are being absorbed in the ileum, the hepatic synthesis of new BAs is inhibited [20]. The importance of the FGF19 regulatory pathway for hepatic BA synthesis has been confirmed by low plasma levels in patients with chronic diarrhoea, suggesting a deficiency in this feedback mechanism as the major cause of excessive BA synthesis-induced diarrhoea [28].

### 3.3. Takeda G-Coupled Receptor 5 (TGR5)

Alongside FXR, TGR5 is considered to be a vital natural BA receptor [29]. It is located in smooth muscle cells, immune cells, and epithelial cells of the intestine and gallbladder [30]. TGR5 has greater expression in the distal ileum and colon [31] and has been shown to inhibit cytokine generation including the production of TNF-alpha [32]. This receptor mediates the effects of BAs on motility and is activated most potently by LCA, followed by DCA, CDCA, and CA [30]. Whilst it is known that TGR5 is essential for maintaining intestinal barrier integrity, there is now emerging evidence that increased TGR5 expression and specific TGR5 mutations have been identified in inflammatory diseases, such as CD [31].

### 3.4. Bile Acid Regulation in CD

CD is a complex chronic inflammatory gastrointestinal disorder with variable age of onset, disease location, and behaviour [33]. In CD, the whole gastrointestinal tract can be affected [34], although in 50% of patients, the most frequent sites of active disease are in the terminal ileum (TI) and colon [35]. Approximately 30% of patients have only small bowel involvement and the remaining 20% of patients have isolated colonic involvement [17]. As the TI is the major site for BA reabsorption and the most common site for CD inflammation, subsequent TI resection results in increased colonic (secondary) BA concentrations as well as increased colonic FXR expression [36]. Activation of FXR reduces ASBT expression, thereby impeding BA reabsorption [37]. The resultant secondary BA in the colon will then stimulate electrolyte and water secretion, which increases motility and shortens the colonic transit time, producing diarrhoea and other gastrointestinal symptoms such as bloating, urgency, and faecal incontinence [38]. Elevated levels of sulphated secondary BA, such as LCA and DCA, can exert detrimental effects on the architecture and function of the colonic epithelium through multiple mechanisms including oxidative DNA damage, inflammation, activation of NF-*k*B, and enhanced cell proliferation [39]. Thus, whilst ileocaecal resection removes the area of local disease, the remaining colon undergoes resultant immunomodulation.

Crohn's disease can further affect the BA enterohepatic circulation by downregulating the main ileal BA uptake transporter, ASBT. Ileal biopsies have revealed significantly lower ASBT expression and BA enterocyte-apical efflux transporter (BCRP) in patients with CD-associated ileitis compared with controls. This can lead to BA malabsorption and subsequent changes to the BA profiles seen in serum and faecal samples of CD patients. Interestingly, this was found in active as well as in remission states of CD, demonstrating that these alterations are irreversible resulting in persistent diarrhoea [40]. This same study found a significantly reduced mRNA expression of FGF19, ASBT, and FXR in treatment-naïve adolescents with Crohn's ileitis. Nolan et al. demonstrated the reduced expression of FGF19 levels in patients with active CD compared with inactive CD and even in patients who had not undergone ileal resection [41, 42]. They found that serum FGF19 levels were inversely correlated with stool frequency and consistency and C-reactive protein in surgery-naïve patients with the ileal disease [41, 43].

## 4. The Role of the Microbiome in CD

Whilst the aetiology of CD is not completely understood, it is thought to be a combination of the environment, the immune system, genetics, and the microbiome [44]. The current theory in CD pathophysiology is thought to involve the inappropriate and ongoing activation of the mucosal immune system driven by the presence of intestinal microbiota [6]. The human gut harbours a complex microbial ecosystem, and deviation away from gut microbial balance may have an impact on host metabolism and resultant CD [45]. Current research suggests that the luminal bacterial community participates in the initiation and perpetuation of chronic intestinal inflammation with inflammation present in parts of the gut containing the highest bacterial concentrations [46].

The healthy adult gut microbiota is dominated by two phyla, *Firmicutes* and *Bacteroidetes* [6], which comprise 90% of all bacterial species in the gut [47]. Seksik et al. demonstrated that approximately 30% of the dominant bacteria in the typical CD microbiome belonged to new phylogenetic groups not typically dominant in healthy individuals [48]. Compared with healthy controls, Frank et al. reported that in mucosal biopsies of CD patients, there was a decrease in the abundance of 16S rRNA sequences of *Firmicutes* and *Bacteroidetes* with an increase in *Proteobacteria* and *Actinobacteria* [49, 50]. Studies have further demonstrated an increase in adherent-invasive *Escherichia coli* (AIEC) [51] and a positive correlation between CD and an abundance in *Enterobacteriaceae* [48, 52], *Pasteurellaceae* (*haemophilus sp*), *Veillonellaceae*, *Neisseriaceae,* and *Fusobacteriaceae* [53]. There is also a reduction of several genera including *Faecalibacterium*, *Roseburia*, *Blautia*, *Ruminococcus*, *Coprococcus,* and several taxa within the families of *Ruminococcaceae* and *Lachnospiraceae* [53]. Further studies demonstrated a reduction in faecal *Lactobacilli* and *Bifidobacteria* in CD patients [54]. Specific species like *Dialister invisus* and *Clostridioides* were also found to be in lower quantity compared with healthy controls [55].

### 4.1. Adherent-Invasive *Escherichia coli* (AIEC)

Several studies have shown AIEC to be increasingly prevalent in CD patients compared with control patients [50, 56–58]. AIEC is enriched in the ileal mucosa and has the ability to adhere to and invade enterocytes and replicate within macrophages without causing host cell death [59]. Whilst the majority of studies have observed an increase in AIEC only in the ileum, Martinez-Medina et al. demonstrated increased levels in both the ileum and colon, which may be a result of host and/or environmental factors [60]. However, AIEC is rarely found in colon tissues of CD patients and has not been identified in UC patients, suggesting that AIEC has a critical role in the occurrence of ileal Crohn's [58, 61, 62].

In postoperative CD patients, Neut et al. demonstrated the presence of *E coli* was greater at three months than at one year in patients with endoscopic recurrence, implying that this organism may play a role in initiating new lesions [63]. Interestingly, antibodies to the *E coli* membrane C and the CD-associated bacterial sequence I2 have been shown to be associated with small bowel involvement, disease severity, rapid disease progression, and the increased need for surgical intervention [64].

### 4.2. *Fusobacteria*

*Fusobacteria* are strongly proteolytic Gram-negative anaerobes [63]. *Fusobacterium* is a well-known pro-inflammatory bacterium that has been isolated in many patients with CD [65–67]. When investigating microbial changes in postoperative CD patients, Neut et al. demonstrated the presence of *Fusobacteria* strains to be associated with early recurrence [63]. Furthermore, *Fusobacteria* has recently shown to promote the progression of colorectal cancer, a long-term complication of IBD [66]. Whilst the specific mechanism by which *F nucleatum* promotes CD development is unclear, Cao et al. have recently proposed its involvement in activating the endoplasmic reticulum stress pathway during CD development to promote intestinal mucosal barrier destruction [68].

### 4.3. *Faecalibacterium prausnitzii*

*Faecalibacterium prausnitzii* is one of the most abundant human gut bacteria and is a well-known anti-inflammatory organism that is considered to be both a sensor and marker of health [53, 69]. This bacterial species, along with other closely related *Clostridial* species, are key sources of the short-chain fatty acid butyrate, which is the preferred energy source for colonic epithelial cells and exerts anti-inflammatory and pro-intestinal barrier properties in experimental mouse models [70, 71]. These bacteria secrete a microbial anti-inflammatory molecule that inhibits NF-*k*B activity and reduces interferon-y and IL-17 expression [72]. A relative reduction of *F prausnitzii* is seen in CD [49, 73], and a diminished abundance of these bacteria at the time of ileal resection has been associated with a higher rate of endoscopic recurrence six months postoperatively [74].

### 4.4. *Helicobacter pylori*

This organism is well known for being the causative agent in gastric and duodenal ulceration, and despite many studies, an association between these bacteria and CD has yet to be strongly identified [75]. Studies have instead suggested a protective effect of *H pylori* with CD. Sonnenberg and Genta demonstrated an inverse association of *H pylori* with CD patients (odds ratio (OR): 0.48, 95% confidence interval (CI): 0.27–0.79) with a positive association between *H pylori*-negative gastritis and CD (OR: 11.06, 95%CI: 7.98–15.02) [76]. This was affirmed by Bartels et al.'s large cohort study who found a lower prevalence of CD in *H pylori*-positive than in *H pylori*-negative patients (OR 0.36 [0.17–0.75]) [77]. This protective mechanism of *H pylori* against CD is unknown, although it has been suggested that this organism induces the development of FoxP3^+^ regulatory T cells and impairs dendritic cell maturation, which could contribute to reduced inflammation [78].

## 5. The Role of the Microbiome in Postoperative CD Patients

Several studies dating back since the 1980s have indicated that intestinal lesions predominate in the distal bowel where the intestinal microbiome is in greater abundance, with bacteria in the faecal stream being reported as the culprit for the recurrence of intestinal lesions after surgery [79–82]. Animal studies have demonstrated that the ileocaecal valve prevents microbial reflux into the ileum; thus, resecting this region can result in colonisation of the neo-TI by anaerobic bacteria [63]. A diverting ileostomy prevents this reflux of bacteria, as the faecal stream does not cross between the ileum and the colon. However, once the ileostomy is removed and intestinal continuity is restored by anastomosing the small bowel to the remaining colon, the anaerobic bacteria are once again able to colonise in the neo-TI [63, 82]. In 1991, Rutgeerts et al. demonstrated this important finding with his groundbreaking study that showed that 71% of postoperative patients who had one-step surgery and 100% of patients who were re-anastomosed had a relapse at the anastomosis site within 6 months of restoring the intestinal stream [82]. Their findings strongly support the view that CD recurrence in the neo-TI is dependent on faecal stream.

Since these findings, there have been multiple studies exploring the effect of ileocolonic resection on mucosa-associated microbiota. In 2015, De Cruz et al. explored changes in the microbiota of 12 patients with CD undergoing surgical intervention. They found reduced biodiversity at the time of surgery that increased 6 months postoperatively, although the microbiota was still different to healthy individuals. This study further demonstrated that patients who developed recurrence had a predominance of *Enterococcus*, whereas *Firmicutes* was the predominant bacteria in remission states [83]. These findings corroborated with the study conducted by Rajca et al. who also documented a lower abundance of *Firmicutes*, particularly *F prausnitzii*, in patients who relapsed [84]. Of greater interest, this study found that reduced *F prausnitzii* numbers predicted risk of relapse independently of raised inflammatory markers, suggesting that monitoring patient's microbiota might provide a new diagnostic tool in assessing postoperative relapse and recurrence risk in CD patients.

Wright et al. also demonstrated reduced diversity in postoperative CD patients compared with control samples from ileal specimens. In addition, they found the microbial composition differed significantly between postoperative CD patients when examining mucosal and faecal samples [85]. In this study, they predicted that combining the microbial analysis of the ileal mucosa (which includes the presence of *Proteus* and abundance of *Faecalibacterium*) and smoking status at 6 and 18 months postoperatively can accurately report endoscopic recurrence.

Numerous studies have proposed that disease recurrence and remission are associated with distinct gut microbiota profiles at the time of surgery and postoperative follow-up. Mondot et al. demonstrated that patients who were in remission 6 months postoperatively had more complex and greater organisation with bacterial clusters, whilst those who relapsed had much looser microbiota structure with the major bacterial phylum being *Proteobacteria* [86]. More specifically, patients in remission were associated with bacteria in the genera *Bacteroides plebius*, *Dorea*, *Ruminococcus bromii, Faecalibacterium prausnitzii,* and *Dialister*, and relapse was associated with *Gemmiger formicilis*, *Enterococcus durans,* and *Ruminococcous lactaris* [86]. These findings substantiated earlier studies demonstrating a reduction of *Firmicutes* [74, 83, 84], and an increase in AIEC in patients with recurrent disease [50].

When comparing faecal samples, studies revealed that CD patients before surgery that had microbial communities of *Atopium*, *Corynebacterium, Gemella,* and *Rothia* in their faeces developed postoperative recurrence [87]. Strombeck et al. detected high counts of *Actinobacteria* and low counts of *Alistipes* in the faecal microbiota in patients who relapsed at their 1-year follow-up. In fact, Alistipes was discovered to negatively correlate with the Rutgeerts score [88]. Furthermore, *Fusobacterium* increased and *Bifidobacterium* decreased at 1, 3, and 6 months postoperatively in patients with endoscopic recurrence compared with patients in remission [89]. Hamilton et al. also reported findings of *Enterobacteriaceae* being associated with increased risk of disease recurrence, whilst *Lachnospiraceae* was associated with maintenance of remission [90].

## 6. The Bile Acids-Microbiome Axis and Its Effect on CD

There are greater than 400 bacterial species present in the gut microbiome that influence mucosal immune development, structure, function, and mucosal integrity. Even the most subtle changes in the microbiome can have profound implications for mucosal barrier function and immune response [91]. Initial evidence of the role of bacteria in CD pathogenesis was historically provided by the successful role of antibiotics in CD treatment [92] with additional evidence provided by the success of faecal diversion in preventing disease relapse [82]. There is now established evidence that the gut microbiota plays a key role in initiating and maintaining the mucosal inflammatory response in CD [93]. There also seems to be a direct connection between dysregulation of the gut microbiome and BA dysmetabolism [6]. Dysregulation of the gut microbiota may alter the capacity for BA modification, specifically by defective conjugation, transformation, and desulphation [94]. The amount of bile released into the intestine can alter gut colonisation, such that low levels of BA favour proliferation of Gram-negative bacteria, whilst high levels of BA favour the proliferation of Gram-positive bacteria and reduction of the Gram-negative Bacteroides [21]. Tian et al. recently demonstrated that Gram-positive bacteria are more sensitive to BA than Gram-negative bacteria [95].

Duboc et al. demonstrated that secondary BA have anti-inflammatory properties through the inhibition of interleukin-6 (IL-6) IL-6, IL-1b, and TNF-a and the down-regulation of IL-8 production [6]. This anti-inflammatory ability, however, is lost after 3-OH-sulphation. Duboc et al. further illustrated that in active CD, secondary nonsulphated BA was found in reduced quantity, whilst sulphated forms of LCA were found in increased quantity [6]. Interestingly, LCA has recently been found to show reduced toxicity to bacteria in the caecal microbiome in both in vivo and in vitro models [95]. Thus, there is a strong interplay between the dysregulated microbiota, BA metabolism, and the mucosal immune system that can result in a changed immunological function that triggers the inflammatory response in IBD.

## 7. Targeted Therapy in CD

Over the past two decades, the medical compendium in the treatment for CD has expanded exponentially from corticosteroids, which remain the cornerstone of induction therapy, through immunomodulators, nutritional therapy, biological agents, and novel small molecule therapies. Unfortunately, surgery continues to play a pivotal role in achieving disease control for patients with aggressive disease and recurrence risk postoperatively remains high. With the advent of research exploring the impact of BA and the microbiome on CD development, there is now a demand for developing new therapies targeting these areas.

### 7.1. Probiotics

In the last decade, probiotics have become a focus of interest for treating IBD. Probiotics are live microorganisms containing a mixture of *Bifidobacteria*, *Lactobacilli,* and some nonpathogenic bacteria such as *Escherichia* and *Enterococci* [93]. Their mechanism of action is not well understood but hypothesised to improve the intestinal microbial balance, inhibit microbial pathogen growth, maintain the integrity of the intestinal epithelial barrier by decreasing epithelial permeability, and modulate local and systemic immune responses [93, 96]. Probiotic treatment can downregulate proinflammatory cytokine secretion such as TNF-alpha, IFN-gamma, and directly interfering with NF-*k*B activation [97]. Probiotics can also secrete active metabolites such as butyrate to exert numerous anti-inflammatory and cytoprotective actions, with the thought that the use of these metabolites overcomes the risk of infection associated with the ingestion of large bacteria [98]. Recently, Degirolamo et al. demonstrated in mice how probiotics can modify the gut microbiome to enhance the faecal excretion of BA by reducing its ileal reabsorption and repressing the enterohepatic FXR-FGF15 axis [45]. Further research is needed to determine whether colonisation of the gut microbial community with probiotics can influence the BA enterohepatic circulation by modifying the BA pool and size.

### 7.2. Antibiotics

The use of antibiotics in CD is controversial as the beneficial use is counteracted by their high rate of side effects and tolerability [99]. Multiple randomised controlled trials in patients with active, uncomplicated CD have not demonstrated efficacy with antibiotics [100], with its use currently only recommended in patients with disease complicated by infection or perianal fistulising disease [33]. There are multiple studies that implicate antibiotic use as a risk factor in the pathogenesis of CD by altering the microbial composition [99, 101, 102]. How this might impact on bile acid metabolism is unclear with limited information in the literature, and largely based on animal studies [103].

### 7.3. Faecal Microbial Transplant (FMT)

FMT is the infusion of faeces from a healthy donor into the gastrointestinal tract of the recipient [104]. It is currently being used in the treatment for refractory and recurrent *Clostridioides difficile* infection (rCDI) [105] with greater than 90% cure rates [106]. There has been some recent exciting work looking into reduced bile salt hydrolase (BSH) functionality as a cause of rCDI [107]. BSH is an enzyme produced by most major bacterial divisions of the gut microbiota and is involved in the role of deconjugation. Mullish et al. complemented this work by demonstrating that one of the key mechanisms underlying the efficacy of FMT in rCDI is through the restoration of gut microbial BSH functionality [108]. These authors also demonstrated that successful FMT for rCDI is associated with activating the FXR signalling pathway [109]. These studies have demonstrated potential novel therapies for targeting and prevention CDI.

Evidence for its use in CD, however, is limited and weak with only a small number of controlled trials reported in the literature, with the majority comprising of noncomparative cohort studies [104, 110]. Despite this, a recent systematic review has shown clinical response rates in early follow-up to be higher with multiple FMT treatments than with single FMT. The dose or type of FMT (fresh vs frozen) did not influence clinical outcomes, although delivery of FMT via the upper gastrointestinal route demonstrated higher early efficacy rates of 75–100% compared with lower delivery routes (30–58%). Unfortunately, this difference was not upheld beyond 8 weeks [104]. Although this study demonstrates therapeutic potential using FMT, the results should be interpreted with caution due to the overrepresentation of low methodological quality studies.

In 2020, one of the largest cohort studies investigating FMT in CD patients was published [111]. A total of 214 patients were enrolled and followed up for a median duration of 43 months (interquartile range IQR: 28–59). The principal finding of this study concluded that after one month, 73%, 62%, 76%, and 71% of patients with FMT showed an improvement in symptoms of abdominal pain, haematochezia, fever, and diarrhoea, respectively. A further 50% achieved steroid-free remission after FMT treatment. In their multivariate analysis, long disease duration (>5 years) and moderate-to-severe disease were associated with a poor response to FMT, implying that FMT may be of clinical use in the earlier stages of disease. This study is also noteworthy in that 44% of patients achieved clinical response and 20% of sustained clinical remission until the end of the follow-up period of the study. Despite the encouraging results of this study, there was no comparator group and objective parameters such as endoscopy findings and biomarkers were not used to determine remission.

### 7.4. FXR Agonists

There is ongoing research into developing FXR agonists as potential treatment for IBD as well as for hepatic and metabolic disorders. FXR is activated by BA and regulates gene transcription involved in BA synthesis, transport, and metabolism in the liver and intestine [112]. FXR also controls several genes that protect against intestinal inflammation, intestinal permeability, and bacterial overgrowth [1, 24]. Activation of FXR in the intestinal tract decreases the production of proinflammatory cytokines such as IL1-beta, IL-2, IL-6, TNF-alpha, and IFN-gamma, thereby reducing inflammation and epithelial permeability [23]. This was demonstrated by Gadaleta et al. in mice models where FXR agonists improved intestinal permeability and FXR activation counteracted pro-inflammatory cytokine expression and secretion by enterocytes [24].

Wilson et al. have now demonstrated that patients with CD have reduced FXR activation compared to patients without CD and subsequently exert reduced CYP3A4 activity and FGF19 expression [113]. This is an important finding considering the central role that CYP3A4 plays in the metabolism of the majority of drugs currently being used for CD, including corticosteroids and certain biologics such as tofacitinib [113]. These data suggest that FXR agonists should be explored further as a novel therapeutic strategy for IBD.

### 7.5. Bile Acids and Bile Acid Sequestrants (BAS)

The use of BAs to restore intestinal functionality has already been established with the use of ursodeoxycholic acid (UDCA), which is currently licensed for the treatment in primary biliary cholangitis [114]. UDCA acts as an anti-inflammatory, cytoprotective, and anti-apoptotic signalling molecule [115].

Recent studies have demonstrated the role of BAS in the induction of remission and improvement in CD symptoms. A randomised, double-blind placebo-controlled trial of colesevelam in TI resected CD patients with a diagnosis of BAD demonstrated significant improvements with a reduction in the number of liquid stools and an improvement in stool consistency [116]. Devarakonda et al. also recently showed an improvement in stool frequency in CD patients with colesevelam monotherapy from pre- to post-treatment (median 33/week vs 14/week, *p*=0.038) [117]. In addition, patients taking colesevelam had a reduction in their Crohn's disease activity index (CDAI) from pre- to post-treatment (median 213 vs 118, *p*=0.013) and an improvement in their QoL score via the SF36 survey (pretreatment median score 118 vs post-treatment median score 121, *p*=0.005) [117]. Thus, the restoration of BA signalling is an exciting novel avenue to focus on for future therapeutic target for CD patients. Given the importance of BA in the modulation of the microbiome, it would be interesting to explore whether therapeutic interventions that alter BA composition might potentially enable us to modify the microbiome in the future. Current studies evaluating the impact of BAS on the microbiome are underway and will likely be reported later this year.

## 8. The Future

The prevention of postoperative disease recurrence in CD is a high priority given the morbidity associated with clinical and surgical recurrence and the long-term risk of short gut syndrome that may arise from multiple bowel resections [118]. Although there is a large medical compendium of medications for the treatment of CD, some patients become refractory to standard management, whilst others suffer with adverse side effects or encounter significant long-term complication risks, including risk of immunogenicity, recurrent infections, and malignancy [119, 120]. Moreover, many patients will continue to live with mildly active symptoms and endure a poor QoL despite medical treatment [121].

Studies targeting microbiological changes and BA regulation in the postoperative setting may provide greater insight and ultimately help mediate the underlying disease progression. Thus far, therapeutic approaches aimed at adapting the environment at the mucosal border have been attempted with elemental diets, total parenteral nutrition, surgical diversion of the faecal stream, probiotics, and antibiotics [46]. There is now increasing evidence that BAS may alter the composition of intestinal microbial species and thus potentially reduce IBD recurrence, particularly in postoperative CD patients [122]. Manipulation of the colonic bacteria with this drug may prove to be more effective and better tolerated, considering they are noninvasive, cheaper, and have fewer side effects and long-term complications than the currently licensed IBD medications. Further studies are needed to establish a clear correlation, and there is ongoing research in this particular topic with possible answers in the near future.

## Data Availability

Data sharing is not applicable to this article as no datasets were generated or analysed during the current study.
